# Double-head transformer neural network for molecular property prediction

**DOI:** 10.1186/s13321-023-00700-4

**Published:** 2023-02-23

**Authors:** Yuanbing Song, Jinghua Chen, Wenju Wang, Gang Chen, Zhichong Ma

**Affiliations:** College of Communication and Art Design, University of Shanghai for Science and Technology, Shanghai, China

**Keywords:** Molecular property prediction, Transformer, Deep learning, Residual network

## Abstract

Existing molecular property prediction methods based on deep learning ignore the generalization ability of the nonlinear representation of molecular features and the reasonable assignment of weights of molecular features, making it difficult to further improve the accuracy of molecular property prediction. To solve the above problems, an end-to-end double-head transformer neural network (DHTNN) is proposed in this paper for high-precision molecular property prediction. For the data distribution characteristics of the molecular dataset, DHTNN specially designs a new activation function, beaf, which can greatly improve the generalization ability of the nonlinear representation of molecular features. A residual network is introduced in the molecular encoding part to solve the gradient explosion problem and ensure that the model can converge quickly. The transformer based on double-head attention is used to extract molecular intrinsic detail features, and the weights are reasonably assigned for predicting molecular properties with high accuracy. Our model, which was tested on the MoleculeNet [1] benchmark dataset, showed significant performance improvements over other state-of-the-art methods.

## Introduction

Molecular property prediction refers to the effective identification of molecular properties such as lipophilicity, binding affinity, biological activity, and toxicity [2]. For fields such as drug design [3], materials science [4], and genetic engineering [5], accurate and reliable prediction of molecular properties can accelerate the development process and reduce the development cost. Therefore, molecular property prediction has significant research meaning and application value, and is a popular research at present.

The quantitative structure-activity/property relationship (QSAR/QSPR) has always been a hot topic in materials chemistry [6]. This method uses mathematical and statistical methods to study the quantitative relationship between the chemical structure of a compound and its physicochemical properties in order to build predictive models [7, 8]. The chemical descriptors used in the QSAR/QSPR model must be able to quantitatively represent the structural parameters of the molecule [9]. Therefore, the prediction accuracy of the model is strongly influenced by the chemical descriptors. A large amount of research is needed to calculate the structural parameters of molecules based on physicochemical experiments [10], and there may be large errors.

With the rise of artificial intelligence, combining artificial intelligence with the field of molecular property prediction has become a major research trend for improving the accuracy of molecular property prediction [11–14]. Current research on the prediction of molecular property by artificial intelligence is mainly divided into two categories: machine learning methods and deep learning methods.

### Machine learning methods

Commonly used prediction models are ridge regression, random forest(RF), elastic network, support vector machine(SVM), gradient boosting and extreme gradient boosting (XGBoost). Ridge regression [15] is a regressor that has a kernel with a regularization term, and the model uses the sum of the weighted kernel functions of the molecules to be predicted and all the molecules in the training set for prediction. RF [16] incorporates random attribute selection in the training process and integrates the results of multiple decision tree models as predictions using the bagging integration method. The model is easy to implement, and the computational cost is small. When the chemical descriptor is Morgan fingerprints [17, 18], running Random Forest on Morgan fingerprints [17, 18] can predict molecular property, such as the model RF on Morgan [19]. The elastic network [20] is a linear model that differs from ridge regression by penalizing the mixed regularization term (L1) and the regularization term (L2), with an additional hyperparameter controlling L1 and L2. SVM [21–23] is a class of generalized linear classifiers that perform binary classification of molecular data by supervised learning. The decision boundary is the maximum margin hyperplane for learning samples. It can transform the molecular property prediction problem into solving convex quadratic programming problem. The use of kernel function avoids the dimension disaster, but the selection of kernel function has a great impact on the performance of SVM. Gradient boosting [24, 25] trains the new-joined weak classifier based on the negative gradient information of the loss function from the current molecular property prediction model. In each iteration, a weak classifier will be obtained. These weak classifiers are accumulated to get the final model. However, this form has the disadvantages of bad parallelization, slow computational speed, and high computational complexity. Given the shortcomings of gradient boosting, XGBoost [26, 27] was proposed by improving the loss function and regularization. XGBoost [28, 29] is an integrated tree model containing multiple classification and regression trees (CART); it adds together the corresponding prediction values of each tree to obtain the final prediction value. XGBoost sorts the data before training and saves it as a block structure to achieve parallel computation. CART and linear classifiers can also be supported as base classifiers to speed up training. The method uses the idea of RF to support row down-sampling and column down-sampling. The first- and second-order derivatives are also used in the custom loss function calculations, and regular terms are added. These methods can reduce the error of the model to prevent the overfitting phenomenon and reduce the computational complexity, which can facilitate faster and more accurate gradient descent. In addition, XGBoost can multiply the weights of leaf nodes by the learning rate after one iteration to weaken the influence of each tree and expand the learning range.

Overall, machine learning methods require domain experts to extract features manually, but their handcrafted molecular descriptors are easily limited by the subjective experience and knowledge of the experts.

### Deep learning methods

Unlike machine learning methods, deep learning enables features to be extracted automatically, so deep learning methods are particularly suitable for molecular property prediction. The feed-forward neural network (FFN) is one of the simplest artificial neural network [30]. The neurons in the former layer are only connected with those in the latter layer. FFN reads chemical descriptors to extract molecular features so as to perform prediction of molecular properties, such as the models FFN on Morgan [19], FFN on Morgan Counts [19], and FFN on RDKit [19]. Later, a large number of neural networks emerged, for example, the directed acyclic graph model [31], deep tensor neural network [32] and message passing neural network (MPNN) [33], which can be used to predict molecular properties. Wu et al. [1] integrated these neural networks in the open-source library DeepChem [34]. Experiments were conducted on different datasets in MoleculeNet [1], and the best model was named MolNet [19]. The MPNN was proposed by Gilmer et al. [33] and is a graph-supervised general model framework for molecular property prediction. Its shortcomings are that it is difficult to use when the molecular size is large, and the number of input messages in the established fully connected graph depends on the number of nodes. Withnall et al. [35] introduced the attention block and the edge memory block into the MPNN framework and proposed the attention message passing neural network (AMPNN) model and the edge memory message neural network (EMNN) model. AMPNN and EMNN only need to use the underlying chemical map data, without additional chemical descriptors. The introduction of the attention mechanism in AMPNN makes the model interpretable. While the performance of EMNN is better than that of AMPNN, the computing cost is also higher. Maziarka et al. [36] applied the transformer encoder to molecules and proposed the molecule attention transformer (MAT) model. The attention mechanism in transformer is strengthened through the distance between atoms and the molecular graph structure. However, the lack of features obtained by the model limits the improvement of the model performance. Furthermore, Wang et al. [37] used graphs to represent molecular data, using vectors to represent atoms and representing each molecule as a matrix according to the connections between atoms. In addition, to preserve the spatial connectivity information on molecules, a convolutional spatial graph embedding layer (C-SGEL) is introduced on the graph convolutional neural network, and multiple C-SGELs are stacked to form a convolutional spatial graph embedding network. The network can learn feature representations in molecular graphs while introducing molecular fingerprints to improve the generalization ability of the network. Chen et al. [38] proposed the algebraic graph-assisted bidirectional transformer (AGBT) model to focus on three-dimensional (3D) information for molecules. Algebraic graphs generate low-dimensional molecular representations. Furthermore, the deep bidirectional transformer (DBT) learns the basic principles of molecular composition from datasets. The molecular property prediction task is completed through fine-tuning. There is a large error in fusing these two molecular representations, which are from algebraic graphs and DBT. Moreover, Cho et al. [39] proposed a 3D graph convolution network to extract 3D molecular structures from molecular graphs and combined it with a graph convolution network, which can accurately predict the global and local property of molecules. The method has high generalization ability and is particularly suitable for protein ligand binding affinity prediction. Sun et al. [40] proposed InfoGraph, an unsupervised graph representation learning model, to maximize the mutual information between the representation of the whole graph and the representation of substructures at different scales. Subsequently, it was extended to semi-supervised learning tasks for graph-level representations, and the semi-supervised learning model InfoGraph* was further proposed. InfoGraph* maximizes the mutual information between unsupervised graph representations learned by InfoGraph and those learned by existing supervised methods. InfoGraph is used to train unlabeled data, and supervised learning can also be used to train labeled data. InfoGraph models and InfoGraph* models perform well in graph classification and molecular property prediction, and have enriched the research in the field of semi-supervised learning graph structure data. Meng et al. [41] proposed the extended graph convolution neural network for the construction of new molecular graphs by fusing ideas such as the graph attention network and gated graph neural network. A new molecular graph is constructed from the vertices of the atom groups, and an attention mechanism is added to focus on the atom groups that affect the prediction of molecular properties, making the model interpretable using gated jump connections. However, the model does not have the best performance on all tasks. Hu et al. [42] proposed a pre-trained neural network strategy and a self-supervised approach based on pre-training a graph neural network with expressive power at the level of individual nodes and the whole graph using easily accessible node-level information. This method learns both local and global representations and generates graph-level representations. This strategy, used together with the graph isomorphism network(GIN), can avoid negative migration between downstream tasks and improve the generalization of downstream tasks, but its robustness still needs to be further improved. Liao et al. [43] proposed LanczosNet, a multiscale graph convolution model, for efficient processing of graph structured data. The model is based on the tri-diagonal decomposition of the Lanczos algorithm, which is used to construct a low-rank approximation of the graph Laplacian operator. This method can efficiently calculate matrix powers and collect the multiscale information, and also builds a learnable spectral filter to expand the model capacity. Chen et al. [44] proposed a local relational pool model on the substructure counting to complete the molecular property prediction by considering the existence of substructures at the local level. This method is superior to most models and allows efficient counting of subgraphs and induced subgraphs on random synthetic graphs. In contrast to the GNN variant, it can learn substructure information from the data and does not depend on manual production. Inspired by multi-view learning, Ma et al. [45] proposed a multi-view graph neural network (MV-GNN) considering the information of atoms and bonds. The method includes a shared self-attention readout component to make the model interpretable. In order to enable information communication between two views, the method proposes a cross-dependent information transfer scheme that produces a variant of MV-GNN, MV-GNNcross, which has better expressiveness. Both models have strong robustness. Bécigneul et al. [46] proposed a model for computing graph embeddings using argument prototypes in order to address the problem of the loss of structural or semantic information owing to averaging or summing the embedded nodes into an aggregated graph representation. The method combines a parametric graph model and optimal transport to learn graph representation, which improves the representational power of the model. The model also produces a smoother graph embedding space compared to the common GNN method. Tang et al. [47] proposed a graph neural network framework, which is based on a self-attention message passing neural network, to identify the relationship between lipophilicity and water solubility with structure, and thus study the relationship between the molecular properties and structure. The use of self-attention mechanisms improves the interpretability of the model and enables visualization based on the contribution of each atom to the property. Yang et al. [19] proposed the directed MPNN(DMPNN), which uses a mixed representation of key-centered convolution encoding molecules and descriptors to make the encoding flexible and strongly prioritized, improving the generalization ability. The model obtains excellent performance on both public and private datasets, but the molecular property prediction performance is poor when the model contains 3D information, the dataset is small, or the classes are particularly unbalanced.

In conclusion, we found that the current molecular property prediction based on deep learning techniques has the problem of low prediction accuracy. The main reason for this problem is poor generalization ability due to the use of traditional activation functions, such as ReLU, PReLU, and Tanh, in the nonlinear representation of molecular features. There may be problems with gradient disappearance or explosion in the network. The global information cannot be taken into account when molecular detail features are extracted. In this regard, this paper makes the following contributions.

1. A new neural network framework, DHTNN, is proposed; it uses an activation function (Beaf), residual network, and transformer based on Double-head attention to process and extract molecular features for high-precision molecular property prediction.
2. A new activation function, Beaf, is defined, which can nonlinearize the molecular characteristics. Compared with other activation functions, the performance of our model DHTNN using the activation function beaf is improved.
3. The molecular residual network is introduced to solve the gradient problem of the neural network and ensure that the model can converge quickly.
4. The Transformer based on Double-head attention extracts the intrinsic detailed features of molecules and acquires global information in parallel, effectively improving the performance of the model in predicting molecular properties.
5. Our method was experimentally tested on six datasets from the MoleculeNet [1] benchmark dataset, and achieved better performance compared to current machine learning and deep learning methods.

## Specific method

**Fig. 1:**
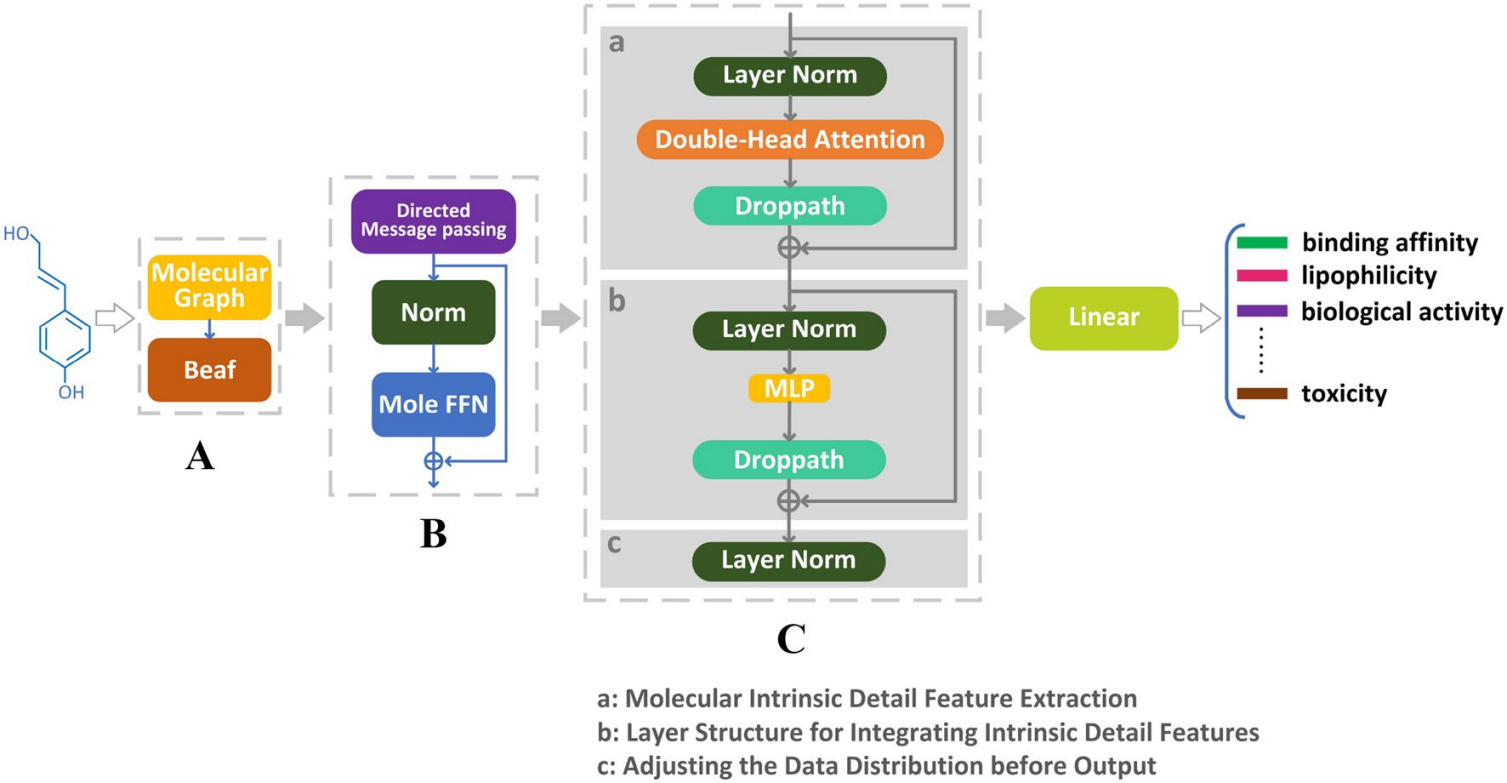
Overall DHTNN architectural diagram. **A** High-precision nonlinear generalization representation of molecular features. **B** Molecular residual network encoding. **C** Molecular feature extraction of Transformer based on Double-head attention

The neural network framework is divided into three parts, as shown in Fig. 1, which are the high-precision nonlinear generalization representation of molecular features, the molecular residual network encoding, and the molecular feature extraction of Transformer based on the Double-head block. The high-precision nonlinear generalization representation of molecular features is used to improve the accuracy and generalization of the algorithm model using a new activation function, Beaf, after the molecular chemical formula is transformed into a molecular map. The molecular residual network encoding part contains the directed MPNN, the batch normalization layer, the molecular feed forward neural network(Mole FFN), and the residual network. Its function is to adjust the data distribution and pass the data forward after encoding the molecular map of the previous section into a matrix. A residual network is added to keep the neural network gradient from disappearing or exploding. The Molecular feature extraction of the Transformer based on the Double-head block quickly and accurately extracts intrinsic detailed features in molecules and obtains molecular global information in parallel to further improve the model prediction performance.

### High-precision nonlinear generalization representation of molecular features

**Fig. 2:**
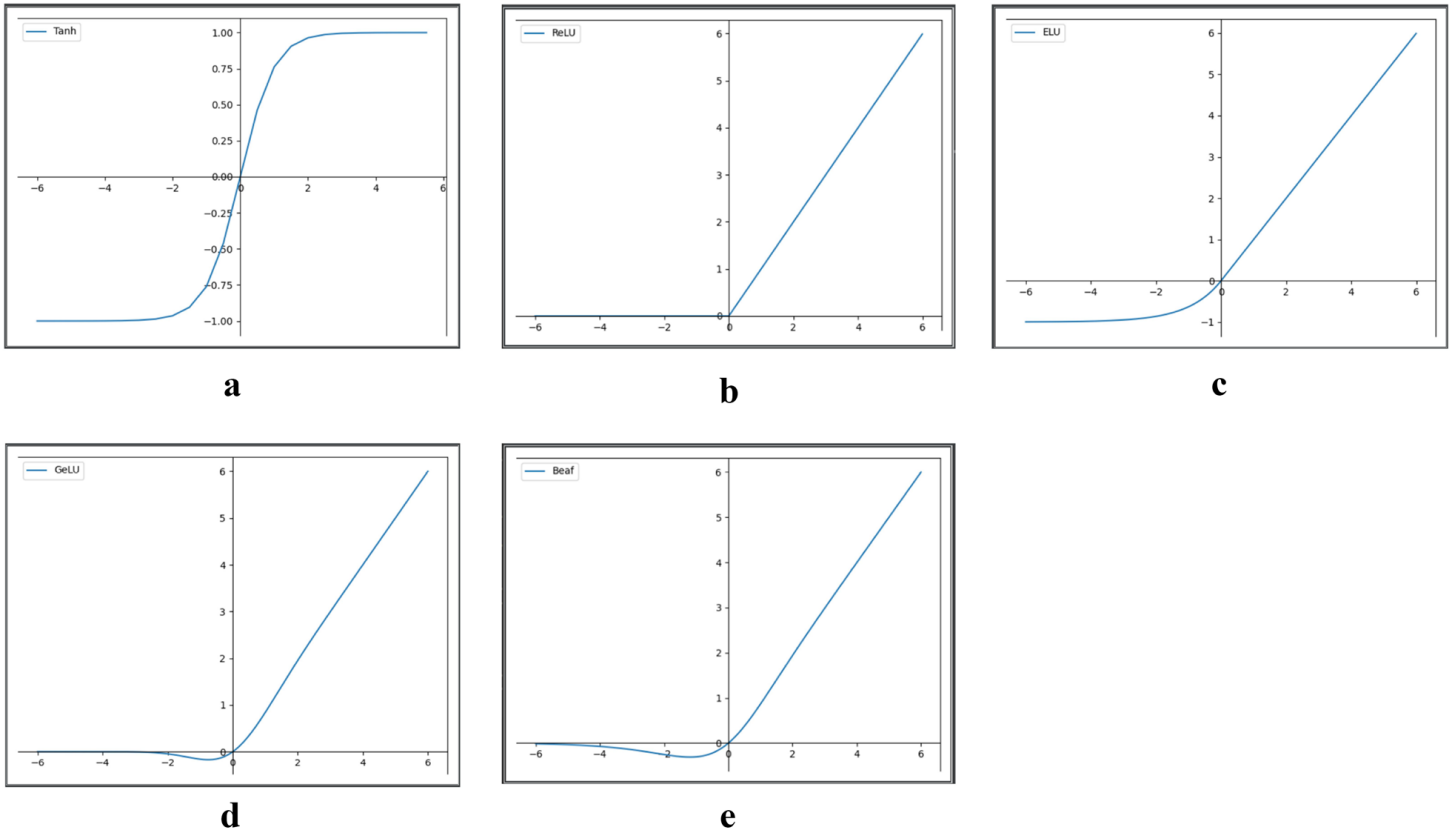
Images of Tanh (**a**), ReLU (**b**), ELU (**c**), GeLU (**d**) and Beaf (**e**)

In this paper, a DHTNN is proposed for molecular property prediction. The molecular residual network encoding structure is proposed in this neural network framework structure, in which a graph convolution neural network is used for the message passing process. Hence, for any molecular dataset, the input molecular chemical formula needs to be first converted into the form of a molecular map. In order to facilitate data reading and memory saving by computers, the molecular chemical formula is usually represented by a matrix [19, 47, 48], which contains atom features and bond features. The input and output of a neural network need to be nonlinear so that the neural network can fit complex functions as the number of layers deepens. By introducing activation functions, neural networks can be equipped with nonlinear characteristics. The commonly used activation function has some shortcomings, such as easy saturation, inability to map the negative value part, or inaccurate mapping of the negative value part, which ultimately makes it difficult to improve the accuracy of molecular property prediction. For example, Tanh approaches saturation at x = 3 (as shown in Fig. 2a). The gradient disappears after saturation. From the ReLU function image (as shown in Fig. 2b), the derivative is one when $x > 0$, and there is no gradient decay. However, the value of the function is constant zero when $x < 0$ and the function cannot complete the accurate mapping, which directly affects the accuracy of nonlinearized molecular features. ELU improves on ReLU for the part of the function that is less than zero. From its function image (as shown in Fig. 2c), it also has the mapping capability in the negative part. However, the curves are flatter and there is little differentiation between values after mapping. The GeLU function image (as shown in Fig. 2d) is smooth, but the function value quickly tends to zero in the negative half-axis. Therefore, the nonlinearized mapping about GeLU is very limited for the part less than zero.

To address the shortcomings of the existing activation functions, such as Tanh is easy to saturate, the negative part of ReLU cannot be mapped, and the negative part of ELU and GeLU are not mapped accurately. In this paper, we propose the activation function Beaf, which is more suitable for molecular feature nonlinearization mapping and has better generalization. The specific equation is as follows:

1 $$\begin{aligned} f\left( x\right) =x\cdot tanh\left( s\left( x\right) \right) -c, \ where \ s(x)=SoftPlus(x)=In(1+e^x) \end{aligned}$$

where *x* denotes the input, and$\ f\left( x\right)$ denotes the output. From Equation (1), Beaf consists of a primary function x, Tanh, SoftPlus and a constant c, which enables a nonlinearized mapping. The function introduces a constant c, $c\in (0,0.004]$. It can adjust the function up and down translation, so as to control the speed of the function value tends to zero, so that the function is more flexible. Combined with our proposed model DHTNN, we take a value of 0.002 for the constant c here. This is because experiments were performed on Lipophilicity, PDBbind, PCBA, BACE, Tox21, and SIDER datasets, and better accuracy of molecular property prediction achieves on all these different datasets when c = 0.002. Thus, it is further demonstrated that Beaf can better nonlinearize the molecular features when c is taken as 0.002. The Beaf function image is shown in Fig. 2e, and in contrast to Tanh (as shown in Fig. 2a), Beaf does not saturate and is derivable everywhere; The negative part can also be mapped compared to ReLU (as shown in Fig. 2b); Compared with ELU (as shown in Fig. 2c), the nonlinear mapping in the negative part is more obvious, the distinction between values after mapping is greater, and the mapping is more accurate; Compared with GeLU (as in Fig. 2d), it does not converge to zero prematurely and is able to map more negative values.

### Molecular residual network encoding

**Fig. 3:**
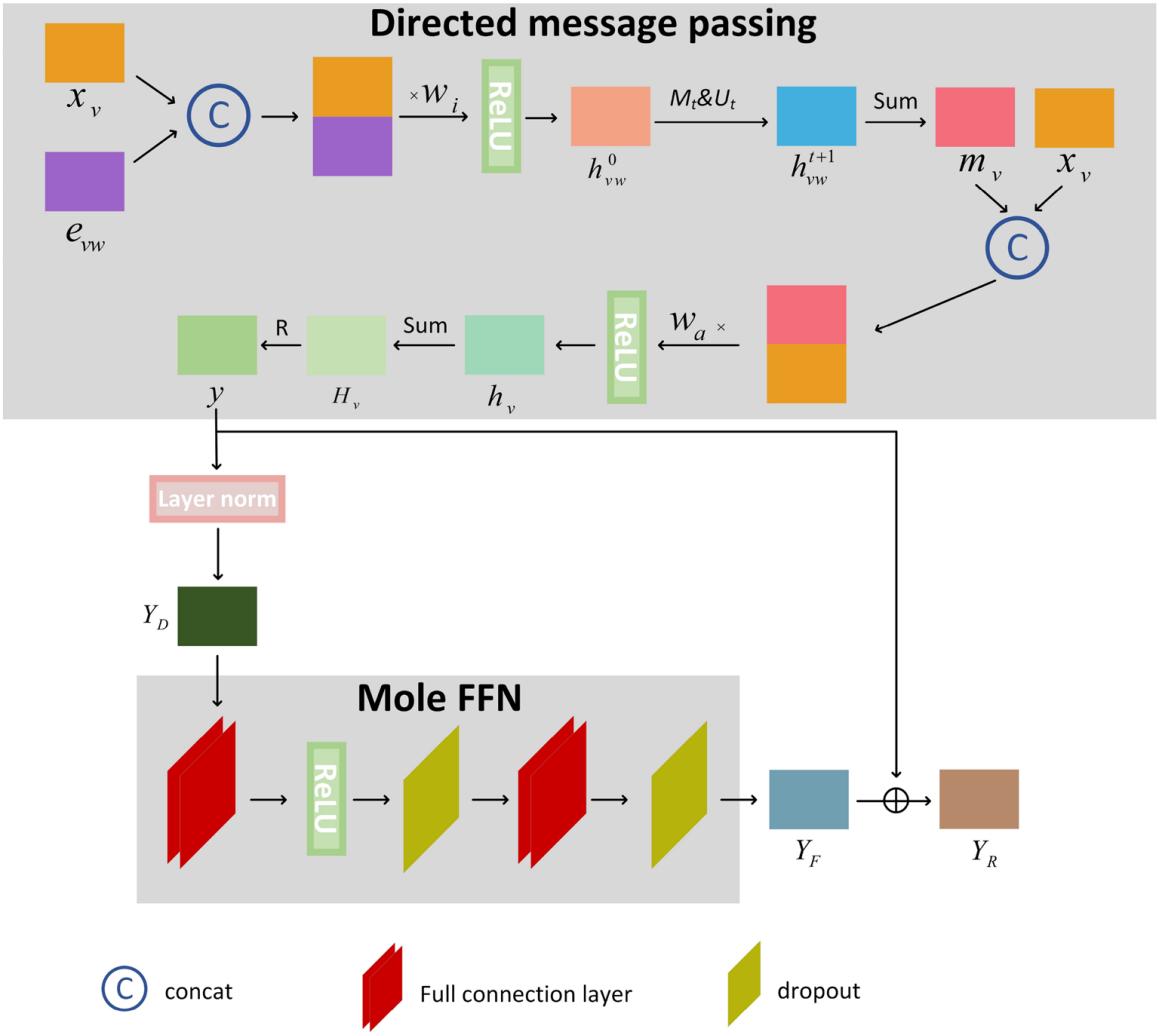
Diagram of the molecular residual network encoding framework. The framework contains a directed MPNN, a batch normalization layer, a molecular feed forward neural network, and a residual network

After the high-precision nonlinear generalization representation of the molecular features in "High-precision nonlinear generalization representation of molecular features" Section is used to obtain the molecular map matrix, the molecular map matrix is subsequently encoded with a molecular residual network (shown in Fig. 3). The specific steps are as follows:

#### Directed MPNN [19]

This acts on the molecular map for encoding. The directed MPNN can be divided into two phases: the directed message passing phase and the readout phase.

The directed MPNN needs to initialize the hidden state of the bond ($h_{vw}^0$) before the message passing phase, as shown in Equation (2).

2 $$\begin{aligned} h_{v w}^{0}=\tau \left( W_{i} {\text {cat}}\left( x_{v}, e_{v w}\right) \right) \end{aligned}$$

where $x_v$ is the node feature, $e_{vw}$ is the edge feature, $W_i$ is the learnable matrix,$\ cat\left( x_v,e_{vw}\right)$ splices the atom feature and the bond feature, and $\tau$ is the activation function ReLU.

This is followed by a directed message passing phase, which contains the message function $M_t$ and the bond update function $U_t$. $M_t$ sends bond-related messages to obtain $m_{vw}^{t+1}$, as shown in Equation (3). Then, $U_t$ updates the hidden state of each bond in the graph to obtain $h_{vw}^{t+1}$, as shown in Equation (4).

3 $$\begin{aligned} m_{v w}^{t+1}= & {} \sum _{k \in \{N(v) \backslash w\}} M_{t}\left( x_{v}, x_{k}, h_{v k}^{t}\right) \end{aligned}$$

4 $$\begin{aligned} h_{v w}^{t+1}= & {} U_{t}\left( h_{v w}^{t}, m_{v w}^{t+1}\right) =\tau \left( h_{v w}^{0}+W_{m} m_{v w}^{t+1}\right) , t \in \{1, \cdots , T\} \end{aligned}$$

where $N(v) \backslash w$ is the neighbourhood edge of the bond *vw* in the graph, and each step of the directed message passing phase is done, for a total of T steps.

The atom hidden state ($h_v$) of the molecule is obtained by summing up the bond hidden states, as shown in Equations (5) and (6).

5 $$\begin{aligned} m_{v}= & {} \sum _{w \in N(v)} h_{v w}^{T} \end{aligned}$$

6 $$\begin{aligned} h_{v}= & {} \tau \left( W_{a} {\text {cat}}\left( x_{v}, m_{v}\right) \right) \end{aligned}$$

We sum $h_v$ to obtain $H_v$, and use the readout function *R* to yield the characteristic *y* of the molecule, as shown in Equations (7) and (8).

7 $$\begin{aligned} H_{v}= & {} \sum _{v \in G} h_{v} \end{aligned}$$

8 $$\begin{aligned} y= & {} R\left( \left\{ H_{v} \mid v \in G\right\} \right) \end{aligned}$$

#### Adjusting data distribution

When training a neural network, the parameters of the previous layer affect the input of the later layer, thus making the training complicated. This requires normalizing the encoded data, adjusting the distribution of the data, reducing the internal covariance bias, and improving the training speed. Therefore, batch normalization is required to optimize the mean position and variance size to make the new data distribution more closely match the real data distribution.

Normalization is done mainly by processing the mean ($E\left[ y\right]$) and variance ($Var\left[ y\right]$) of a batch of data consisting of one layer. In order to calculate the numerical stability, the constant $\epsilon$ is added; the learnable parameters $\gamma$ and $\beta$ are introduced for optimization as a way to improve the nonlinear expression, as shown in Equation (9).

9 $$\begin{aligned} Y_{D}=\frac{y-E[y]}{\sqrt{{\text {Var}}[y]+\epsilon }} * \gamma +\beta \end{aligned}$$

#### Aggregating to generate global features

The molecular feed forward neural network receives the data ($Y_D$) after the batch normalization process for aggregation. The molecular feed forward neural network consists of five layers of network structure: the fully connected layer, activation function, dropout layer, fully connected layer, and dropout layer. The molecular feed forward neural network can aggregate local features into global features, ($Y_F$), which reduces the influence of the feature location on test results, prevents overfitting, and improves the model generalization ability. The implementation process can be characterized as in Equation (10):

10 $$\begin{aligned} Y_{F}= \text{ MoleFFN } \left( Y_{D}\right) \end{aligned}$$

#### Preventing gradients disappearance

As the number of neural network layers deepens, there is a gradual decrease in the accuracy of the training and test sets owing to gradient disappearance and gradient explosion, so the neural network cannot converge. The residual network connection is used after the batch normalization process and the molecular feed forward neural network, and the *y* obtained from the directed MPNN and the $Y_F$ obtained from the molecular feed forward neural network are connected with residuals to obtain $Y_R$. The residual network learns the difference between the input and output, and these two layers do an all-equal mapping to ensure that the gradient problem does not affect the results of the neural network, as shown in Equation (11).

11 $$\begin{aligned} Y_{R}=y \oplus Y_{F} \end{aligned}$$

### Molecular feature extraction of Transformer based on Double-head attention

**Fig. 4:**
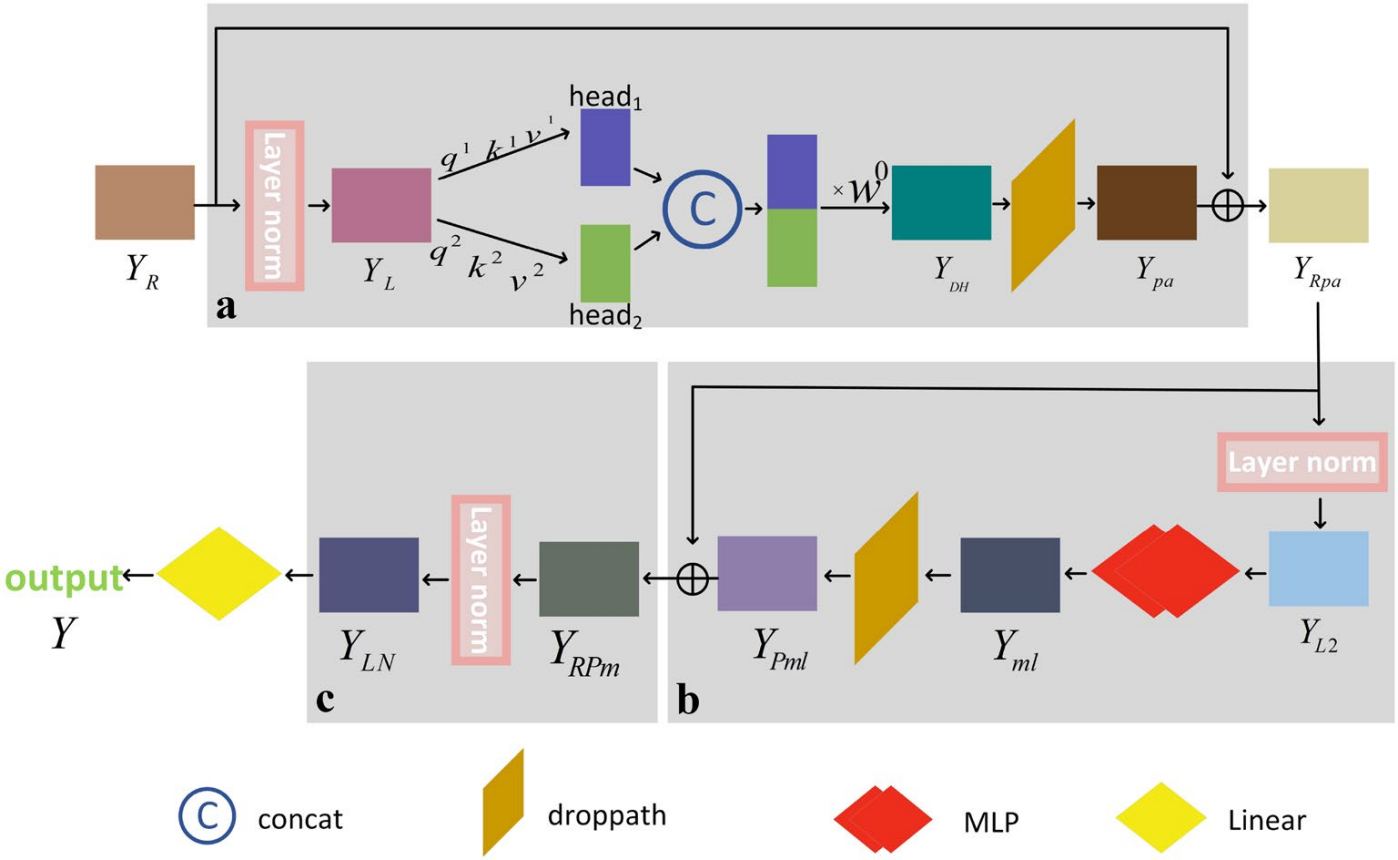
Molecular feature extraction of Transformer based on Double-head attention. **a** Molecular intrinsic detail feature extraction. **b** Layer structure for integrating intrinsic detail features. **c** Adjusting the data distribution before output

The molecular map matrix ($Y_R$) obtained from the molecular residual network encoding is input to the molecular feature extraction of Transformer based on the Double-head attention block for obtaining molecular features (shown in Fig. 4), which contains double-head attention, Multilayer Perceptron (MLP), layer normalization, Droppath, and residual connectivity. Its processing is divided into three main steps:

#### Molecular intrinsic detail feature extraction

The molecular graph matrix is input to the first part of the molecular feature extraction of Transformer based on the Double-head attention block, as shown in Fig. 4a. This part consists of layer normalization, double-head attention, Droppath, and residual connection for extracting the intrinsic detail features in the molecular graph and assigning the weights reasonably.

1. Layer normalization: each data point ($Y_R$) obtained by Equation (11) is normalized to adjust the molecular characteristic distribution. The normalization is processed by calculating the mean, ${E\left[ Y_R\right] }^l$, and the variance, ${Var\left[ Y_R\right] }^l$, of each data point. In order to calculate the stability of the values and prevent the denominator from being zero, the constant $\epsilon$ is added. The learnable parameters $\gamma$ and $\beta$ are introduced as a way to improve the nonlinear expression. The process is shown in Equation (12):
  12 $$\begin{aligned} Y_{L}=\frac{Y_{R}-E\left[ Y_{R}\right] ^{l}}{\sqrt{{\text {Var}} \left[ Y_{R}\right] ^{l}+\epsilon }} * \gamma +\beta \end{aligned}$$
2. Double-head attention: The weights are rationally assigned, increasing the weight of important information and decreasing the weight of unimportant information. This process allows the model to learn relevant information from both spaces. $W^q$, $W^k$, and $W^v$ are three trainable shared matrices. The $Y_L$ obtained by layer normalization is multiplied with $W^q$, $W^k$, and $W^v$ to obtain *q*, *k*, and *v*, respectively. The calculation processes are given in Equations (13, 14, 15).
  13 $$\begin{aligned} q= & {} Y_{L} W^{q} \end{aligned}$$
  14 $$\begin{aligned} k= & {} Y_{L} W^{k} \end{aligned}$$
  15 $$\begin{aligned} v= & {} Y_{L} W^{v} \end{aligned}$$
  As the molecular graph matrix only has the information of the length and width, this paper proposes Double-head attention to extract the information of the length and width of the molecular graph matrix; that is $head = 2$, so *q*, *k* and *v* are divided into two parts. *q* is split into $q^1$ and $q^2$. *k* is split into $k^1$ and $k^2$. *v* is split into $v^1$ and $v^2$. Then, $q^1$, $k^1$ and $v^1$ belong to ${\ head}_1$. $q^2$, $k^2$ and $v^2$ belong to ${\ head}_2$. ${\ head}_1$ and ${\ head}_2$ are calculated as shown in Equations (16, 17), where $d_{k^1}$ and $d_{k^2}$ are the dimensions of $k^1$ and $k^2$, respectively.
  16 $$\begin{aligned} {\text {head}}_{1}= & {} {\text {Attention}}\left( q^{1}, k^{1}, v^{1}\right) ={\text {softmax}}\left( \frac{q^{1} k^{1^{T}}}{\sqrt{d_{k^{1}}}}\right) v^{1} \end{aligned}$$
  17 $$\begin{aligned} {\text {head}}_{2}= & {} {\text {Attention}}\left( q^{2}, k^{2}, v^{2}\right) ={\text {softmax}}\left( \frac{q^{2} k^{2^{T}}}{\sqrt{d_{k^{2}}}}\right) v^{2} \end{aligned}$$
  The output ($Y_{DH}$) of Double-head attention (DoubleHead) is obtained by concatenating ${\ head}_1$ and ${\ head}_2$ together, and the calculation formula is given in Equation (18). Here, $W^{o}$ is the parameter matrix for better fusion of the concatenated data and ensures that the vector lengths of the input and output of DoubleHead remain unchanged.
  18 $$\begin{aligned} Y_{D H}= \text{ DoubleHead } (q, k, v)= \text{ Concat } \left( \text {head}_{1}, \text {head}_{2}\right) W^{o} \end{aligned}$$
3. Droppath: This contains two types of droppings. One is local dropping, and the other is global dropping. Local dropping means dropping layers randomly with a certain probability, but it is guaranteed that one branch must be through. Global dropping randomly selects a branch and discards the rest of the layers. The two types of droppings are alternated during the network training [49]. A Droppath operation is performed on $Y_{DH}$, which is obtained in the above double-head attention to obtain $Y_{pa}$, as shown in Equation (19).
  19 $$\begin{aligned} Y_{p a}= \text{ Droppath } \left( Y_{D H}\right) \end{aligned}$$
4. Residual connection: Residual connection is done for the data ($Y_{pa}$) obtained after Droppath, with $Y_R$ obtained from the molecular residual network encoding, as shown in Equation (20).
  20 $$\begin{aligned} Y_{R p a}=Y_{R} \oplus Y_{p a} \end{aligned}$$

#### Layer structure for integrating intrinsic detail features

The extracted intrinsic detail features are integrated and used to output the final molecular property prediction results. The composition structure is similar to that in part a. The only difference is that the double-head attention in part a is replaced by the MLP, as shown in Fig. 4b. The calculation equations are given in Equations (21, 22, 23 and 24) as follows:

21 $$\begin{aligned} Y_{L 2}= & {} \frac{Y_{R p a}-E\left[ Y_{R p a}\right] ^{l}}{\sqrt{{\text {Var}}\left[ Y_{R p a}\right] ^{l}+\epsilon }} * \gamma +\beta \end{aligned}$$

22 $$\begin{aligned} Y_{m l}= & {} M L P \left( \textrm{Y}_{\textrm{L} 2}\right) \end{aligned}$$

23 $$\begin{aligned} Y_{P m l}= & {} \text{ Droppath } \left( Y_{m l}\right) \end{aligned}$$

24 $$\begin{aligned} Y_{R P m}= & {} Y_{R p a} \oplus Y_{P m l} \end{aligned}$$

#### Adjusting the data distribution before output

After the Transformer based on the Double-head attention block, the distribution of data causes large changes, so before outputting the results, layer normalization is performed again, as shown in Fig. 4(c), to adjust the data distribution before output. The calculation formula is shown in Equation (25).

25 $$\begin{aligned} Y_{L N}=\frac{Y_{R P m}-E\left[ Y_{R P m}\right] ^{l}}{\sqrt{{\text {Var}}\left[ Y_{R P m}\right] ^{l}+\epsilon }} * \gamma +\beta \end{aligned}$$

The results of the final molecular property prediction are obtained from the linear layer, as shown in Equation (26).

26 $$\begin{aligned} \textrm{Y}= \text{ Linear } \left( Y_{L N}\right) \end{aligned}$$

## Experiment and discussion

### Sources of experiment molecular datasets and evaluation metrics

#### Dataset Source

In deep learning, datasets play a pivotal role in training the model and verifying the generalization of the proposed algorithm. The dataset used in this paper is from the MoleculeNet [1] benchmark dataset. Six datasets (i.e., Lipophilicity, PDBbind, PCBA, BACE, Tox21, and SIDER) were selected for the task type, including regression and classification, covering three domains (i.e., physiology, physical chemistry, and biophysics). The datasets were divided into a training set, validation set, and test set in the ratio of 8:1:1 with random and scaffold splitting. The training set was used to train the model, the validation set was used to adjust hyperparameters and optimize the model, and the test set was used to evaluate the model performance. At the minimum, the dataset comprises 168 molecules, while the maximum was 437,928 molecules to ensure that the algorithm was applicable to datasets of various sizes.

1. Lipophilicity [50] Lipophilicity is derived from the ChEMBL database, containing 4,200 compounds. The value of lipophilicity was obtained experimentally and calculated by the octanol/water partition coefficient. Lipophilicity affects the membrane permeability and aqueous solubility; therefore, the prediction of lipophilicity is crucial in drug discovery.
2. PDBbind [51–53] PDBbind is a protein-ligand complex binding affinity dataset that establishes a PDB-wide connection between structural and energetic information of protein-ligand complexes.
3. PCBA [54] PubChem BioAssay (PCBA) is a dataset of biological activity; it is generated through high-throughput screening, with 128 bioassays that measure 400,000 compounds.
4. BACE [55] BACE is a dataset of inhibitors of human $\beta$-secretase 1 (BACE-1) containing quantitative (IC50) and qualitative (binary label) results combined with data for 1,513 compounds.
5. Tox21 [56] Toxicology in the 21st Century created the toxicity data collection system, known as the Tox21 dataset, which is a toxicity dataset containing 8,014 compounds.
6. SIDER [57, 58] The Side Effect Resource (SIDER) is a database of listed drugs and adverse drug reactions (ADRs), containing data on 1,427 compounds. It is divided into 27 classes of compounds, with drug side effects according to the organ class.

#### Algorithm evaluation metrics

We tested our neural network framework on six datasets, including two regression datasets (Lipophilicity, PDBbind) and four classification datasets (PCBA, BACE, Tox21, SIDER). The algorithm evaluation metric for the regression dataset was the root mean square error (RMSE), which is the arithmetic square root of the expected value of the squared difference between the parameter estimate and the true value of the parameter. A smaller RMSE indicates a smaller error and better prediction performance. The algorithm evaluation metrics for classification datasets were the area under the recall curve (PRC-AUC) and the area under the receiver operating characteristic curve (ROC-AUC) [59]. Larger AUC values indicate more stable models and better prediction performance.

### Experiment results and analysis

#### Validation of activation function selection

In order to verify the algorithmic effectiveness of our proposed activation function Beaf on our model, we performed validation experiments on the activation function selection. On the six datasets (i.e., Lipophilicity, PDBbind, PCBA, BACE, Tox21 and SIDER), we applied the activation functions Beaf, ELU and GeLU to our algorithmic model and compared their performances, shown in Tables 1 and 2, respectively.

**Table 1:** Comparisons of performance for the activation functions Beaf, ELU, and GeLU on Lipophilicity and PDBbind datasets (lower values are better)

	GeLU	ELU	Beaf
Lipophilicity	$0.635\pm 0.040$	$0.723\pm 0.037$	$0.577\pm 0.049$
PDBbind	$2.019\pm 0.278$	$2.054\pm 0.265$	$1.771\pm 0.300$

**Table 2:** Comparisons of performance for the activation functions Beaf, ELU, and GeLU on PCBA, BACE,Tox21 and SIDER datasets (higher values are better)

	GeLU	ELU	Beaf
PCBA	$0.806\pm 0.002$	$0.663\pm 0.006$	$0.821\pm 0.005$
BACE	$0.928\pm 0.019$	$0.909\pm 0.022$	$0.923\pm 0.035$
Tox21	$0.843\pm 0.025$	$0.840\pm 0.049$	$0.847\pm 0.015$
SIDER	$0.652\pm 0.027$	$0.628\pm 0.012$	$0.679\pm 0.015$

The Lipophilicity and PDBbind datasets, shown in Table 1, are regression datasets. RMSE was used to evaluate our algorithm performance based on these two datasets. A lower RMSE value indicates better performance. As can be seen from Table 1, the RMSE value for our algorithmic model based on the Beaf on the Lipophilicity dataset is $0.577\pm 0.049$, which is 0.146 lower than the $0.723\pm 0.037$ obtained by the ELU. It is also 0.058 lower compared to using the GeLU (GeLU: $0.635\pm 0.040$). On the PDBbind dataset, the RMSE value for our algorithmic model based on the Beaf is $1.771\pm 0.300$, which is 0.283 lower compared to using the ELU (ELU: $2.054\pm 0.265$). It is also 0.248 lower than the $2.019\pm 0.278$ obtained by the GeLU. Therefore, there are significant advantages to use Beaf on the Lipophilicity and PDBbind datasets.

In Table 2, the PCBA, BACE, Tox21 and SIDER datasets are classification datasets. AUC was used to evaluate our algorithm performance based on these four datasets. A higher AUC value indicates better performance. As can be seen from Table 2, the AUC value for our algorithm model based on the Beaf is $0.821\pm 0.005$ on the PCBA dataset. This represents an improvement in the AUC value of 0.158 over the model with ELU (ELU: $0.663\pm 0.006$) and of 0.015 over the model with GeLU (GeLU: $0.806\pm 0.002$). On the BACE dataset, the AUC value for our algorithmic model based on the Beaf is $0.923\pm 0.035$. This represents an improvement in the AUC value of 0.014 over the $0.909\pm 0.022$ obtained by the ELU. This is slightly lower, by 0.005, than the model with the GeLU (GeLU: $0.928\pm 0.019$). On the Tox21 dataset, the AUC value for our algorithmic model is $0.847\pm 0.015$ based on the Beaf. This represents an increase in the AUC value of 0.007 over the $0.840\pm 0.049$ gained by the ELU. It represents an increase in the AUC value of 0.004 compared to using the GeLU (GeLU:$0.843\pm 0.025$). On the SIDER dataset, the AUC value for our algorithmic model based on the Beaf is $0.679 \pm 0.015$. This represents an improvement in the AUC value of 0.051 over the $0.628\pm 0.012$ obtained by the ELU. It represents an increase the AUC value of 0.027 compared to the model with GeLU (GeLU: $0.652\pm 0.027$). Therefore, there are significant advantages of using Beaf on PCBA, BACE, Tox21, and SIDER datasets.

In conclusion, for ELU, all experimental results based on the Beaf are better than those based on the ELU on the datasets Lipophilicity and PDBbind. For GeLU, on the four datasets (i.e., PCBA, BACE, Tox21, and SIDER), only on the BACE dataset, the experimental results based on the GeLU are slightly better than those based on the Beaf. The experimental results of the algorithmic model based on the Beaf are better than those of the algorithmic model based on the GeLU on three of the four datasets. Therefore, we chose Beaf as the activation function for the double-head transformer neural network (DHTNN) for molecular property prediction.

#### Comparison of model performance

Our experiments were run on a Windows 10 operating system with a 1.70 GHz Intel Xeon Bronze 3104 CPU, 64 GB of RAM, and an NVIDIA RTX2080 GPU, using python 3.8 as the development language and PyTorch 1.5.1 as the neural network framework for deep learning training.

The results of our algorithm were compared with the following state-of-the-art methods: MolNet [1], RF on Morgan [19], FFN on Morgan [19], FFN on Morgan counts [19], FFN on RDKit [19], and DMPNN [19]. The chemical descriptors used by RF on Morgan and FFN on Morgan are Morgan fingerprints [17, 18]. FFN on Morgan counts uses count-based Morgan fingerprints. FFN on RDKit uses the chemical descriptors generated by RDKit [60]. The chemical descriptors of MolNet, DMPNN, and our model (DHTNN) are SMILES [61, 62].

The methods used for performance comparison included machine learning methods and deep learning methods, and RF on Morgan is currently the most advanced method for machine learning. MolNet, FFN on Morgan, FFN on Morgan Counts, FFN on RDKit and DMPNN are current advanced methods for deep learning.

**Table 3:** Comparisons of performance with state-of-the-art methods on regression datasets, splitting the datasets by random splitting in a ratio of 8:1:1 (lower values are better)

Methods	Lipophilicity	PDBbind
MolNet [1]	$0.655\pm 0.036$	$1.920\pm 0.070$
RF on Morgan [19]	$0.823\pm 0.035$	$2.083\pm 0.324$
FFN on Morgan [19]	$0.928\pm 0.044$	$2.778\pm 0.599$
FFN on Morgan counts [19]	$0.874\pm 0.043$	$2.901\pm 0.812$
FFN on RDKit [19]	$0.735\pm 0.039$	$2.020\pm 0.376$
DMPNN [19]	$0.582\pm 0.024$	$1.945\pm 0.298$
Ours	${{ {0.577}}}\pm {{ {0.049}}}$	${{ {1.771}}}\pm {{ {0.300}}}$

**Table 4:** Comparisons of performance with state-of-the-art methods on classification datasets, splitting the datasets by random splitting in a ratio of 8:1:1 (higher values are better)

Methods	PCBA	BACE	Tox21	SIDER
MolNet [1]	$0.136 \pm 0.004$	/	$0.829 \pm 0.006$	$0.648 \pm 0.009$
RF on Morgan [19]	/	$0.825 \pm 0.039$	$0.619 \pm 0.015$	$0.572 \pm 0.007$
FFN on Morgan [19]	$0.263 \pm 0.008$	$0.873 \pm 0.040$	$0.788 \pm 0.017$	$0.652 \pm 0.010$
FFN on Morgan Counts [19]	$0.268 \pm 0.006$	$0.882 \pm 0.030$	$0.790 \pm 0.020$	$0.638 \pm 0.020$
FFN on RDKit [19]	$0.207 \pm 0.005$	$0.858 \pm 0.034$	$0.832 \pm 0.016$	$0.654 \pm 0.019$
DMPNN [19]	$0.769 \pm 0.010$	$0.892 \pm 0.031$	$0.839 \pm 0.022$	$0.657 \pm 0.016$
Ours	${{ {0.821}}} \pm {{ {0.005}}}$	${{{0.923}}} \pm {{ {0.035}}}$	${{ {0.847}}} \pm {{{0.015}}}$	${{ {0.679}}} \pm {{ {0.015}}}$

**Fig. 5:**
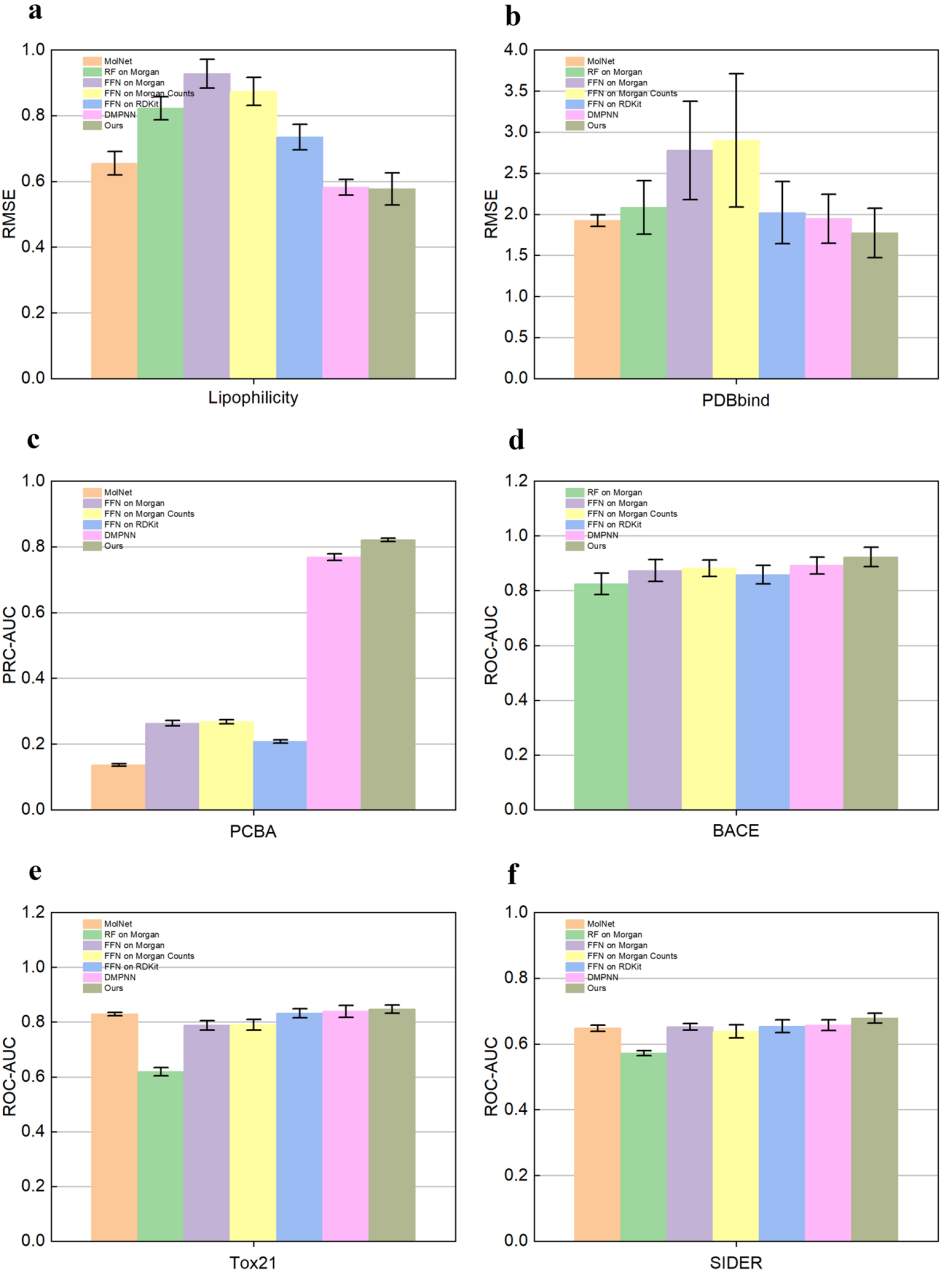
Performance of the model on Lipophilicity (**a**), PDBbind (**b**), PCBA (**c**), BACE (**d**), Tox21 (**e**) and SIDER (**f**) datasets. RMSE was calculated on Lipophilicity (**a**), PDBbind (**b**), the lower the RMSE, the better the model performance. PCBA (**c**), BACE (**d**), Tox21 (**e**), and SIDER (**f**) on which AUC was calculated; the higher the AUC, the better the model performance. Datasets were split by random

**Table 5:** Comparisons of performance with state-of-the-art methods on regression datasets, splitting the datasets by scaffold splitting in a ratio of 8:1:1 (lower values are better)

Methods	Lipophilicity	PDBbind
MolNet [1]	$0.655\pm 0.036$	$1.920\pm 0.070$
RF on Morgan [19]	$0.908\pm 0.052$	$2.011\pm 0.240$
FFN on Morgan [19]	$1.045\pm 0.042$	$2.737\pm 0.518$
FFN on Morgan Counts [19]	$1.003\pm 0.068$	$3.015\pm 0.636$
FFN on RDKit [19]	$0.792\pm 0.032$	$1.842\pm 0.252$
DMPNN [19]	$0.648\pm 0.057$	$1.858\pm 0.300$
Ours	${{ {0.590}}}\pm {{ {0.038}}}$	${{{1.599}}}\pm {{ {0.199}}}$

**Table 6:** Comparisons of performance with state-of-the-art methods on classification datasets, splitting the datasets by scaffold splitting in a ratio of 8:1:1 (higher values are better)

Methods	PCBA	BACE	Tox21	SIDER
MolNet [1]	$0.136 \pm 0.004$	/	$0.829 \pm 0.006$	$0.648 \pm 0.009$
RF on Morgan [19]	/	$0.804 \pm 0.035$	$0.582 \pm 0.031$	$0.540 \pm 0.013$
FFN on Morgan [19]	$0.189 \pm 0.005$	$0.843 \pm 0.052$	$0.722 \pm 0.041$	$0.608 \pm 0.035$
FFN on Morgan Counts [19]	$0.195 \pm 0.003$	$0.849 \pm 0.047$	$0.725 \pm 0.052$	$0.595 \pm 0.033$
FFN on RDKit [19]	$0.161 \pm 0.005$	$0.833 \pm 0.046$	$0.788 \pm 0.046$	$0.618 \pm 0.031$
DMPNN [19]	$0.707 \pm 0.002$	$0.759 \pm 0.0291$	$0.779 \pm 0.037$	$0.602 \pm 0.024$
Ours	${{ {0.715}}} \pm {{ {0.004}}}$	${{ {0.774}}} \pm {{ {0.014}}}$	$0.772 \pm 0.023$	${{ {0.661}}} \pm {{ {0.046}}}$

**Fig. 6:**
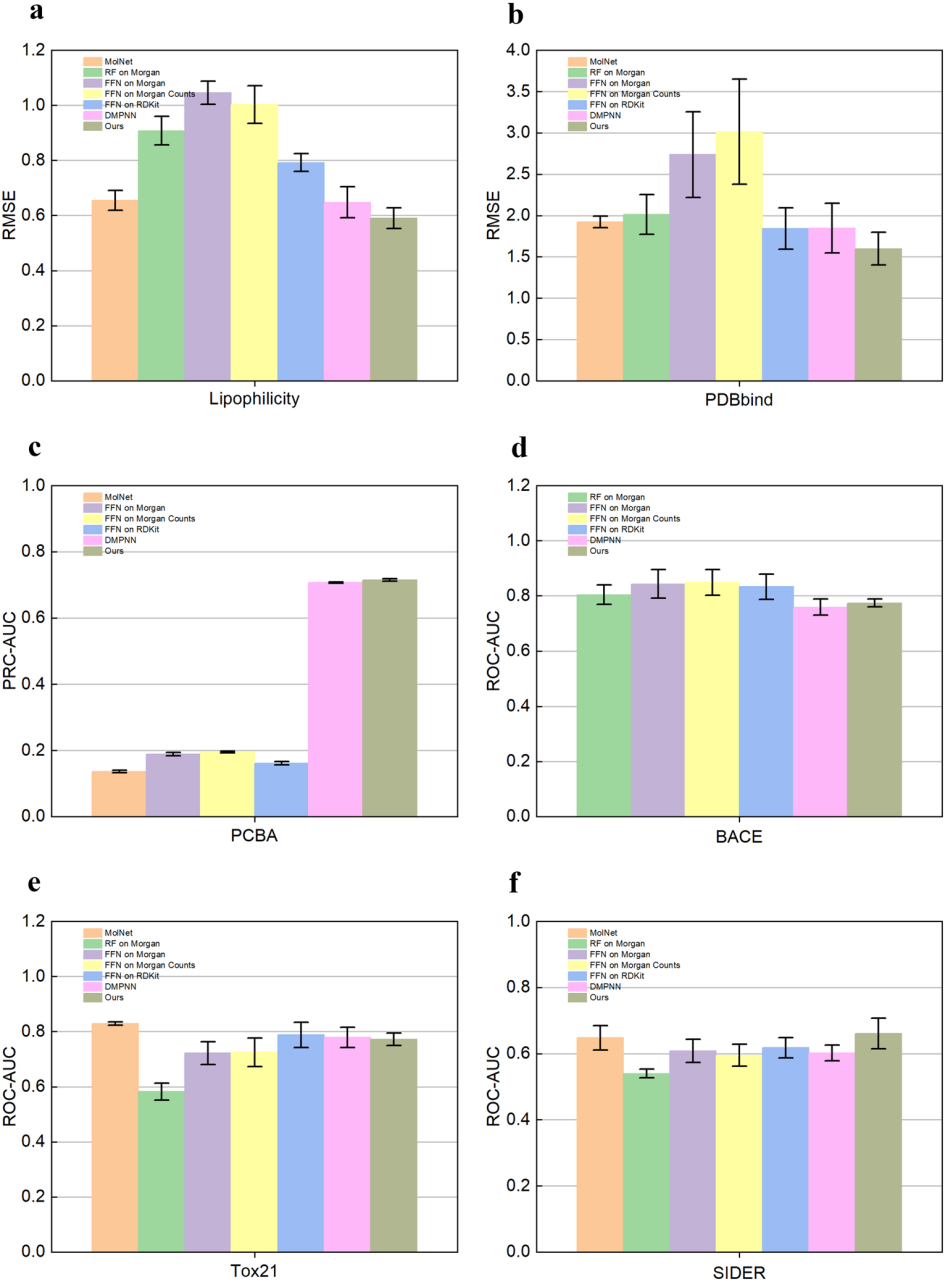
Performance of the model on Lipophilicity (**a**), PDBbind (**b**), PCBA (**c**), BACE (**d**), Tox21 (**e**) and SIDER (**f**) datasets. RMSE was calculated on Lipophilicity (**a**), PDBbind (**b**), the lower the RMSE, the better the model performance. PCBA (**c**), BACE (**d**), Tox21 (**e**), and SIDER (**f**) on which AUC was calculated; the higher the AUC, the better the model performance. Datasets were split by scaffold

For the regression dataset, we calculated the RMSE to evaluate the performance of the algorithm. The lower the RMSE, the better the model performance. As shown in Figs. 5a, b and 6a, b, our model’s RMSE is lower compared to the other models, whether by random splitting or by scaffolding splitting. On the Lipophilicity dataset, our model’s performance (Ours: $0.577 \pm 0.049$) is 0.5% lower compared to DMPNN (DMPNN: $0.582 \pm 0.024$) by random splitting (Table 3). Our model performance (Ours: $0.590 \pm 0.038$) is by 5.8% lower compared to DMPNN (DMPNN: $0.648 \pm 0.057$) by scaffold splitting (Table 4). This is because we use our proposed activation function Beaf in the high-precision nonlinear generalization representation of molecular features. DMPNN uses the activation function ReLU, and the negative part of ReLU is mapped to zero, while Beaf is still able to map the negative part, especially the values between $-4$ and 0. The negative values in the Lipophilicity are concentrated between $-2$ and 0, and after the nonlinear transformation by the Beaf activation function, the neurons in the negative part do not die. Therefore, our model outperforms DMPNN on the regression dataset.

For the classification dataset, we calculated the PRC-AUC and ROC-AUC. The higher the AUC, the better the model performance. As shown in Figs. 5c, d, e, f and 6c, d, e, f all of our models outperform the other models by random splitting. Our model also outperforms the other models on three of the four datasets by scaffold splitting. Only on the Tox21 dataset, the experimental results are slightly worse than those of other models. Compared with the random splitting approach, the scaffold splitting approach provides a more realistic estimation of the model performance. On the PCBA dataset, our model (Ours: $0.821 \pm 0.005$) improves 61.4% compared to FFN on RDkit (FFN on RDkit: $0.207 \pm 0.005$) by random splitting (Table 5). Also, our model (Ours: $0.715 \pm 0.004$) improves by 55.4% compared to FFN on RDkit (FFN on RDkit: $0.161 \pm 0.005$) by scaffold splitting (Table 6). The performance improvement is most significant on the PCBA dataset among all classified datasets. The molecular feature extraction of Transformer based on the Double-head block added to our model is used to learn individual molecular features and atom-to-atom interrelationships. The greater the number of data samples, the richer the intrinsic features learned and the better the molecular property prediction. The PCBA contains 430,000 data samples and is the largest dataset in the four classification datasets used in our experiments. Therefore, the performance improvement of our algorithm is the greatest.

Whether on regression or classification datasets, our model did not exhibit gradient disappearance or explosion. The molecular residual network encoding in the model played an important role in ensuring that the model converged.

## Conclusion

In this paper, a new algorithmic framework, DHTNN, was proposed for molecular property prediction. Beaf, a new activation function, is included in the molecular nonlinear representation part, and the negative part is also able to be mapped, making the mapping more accurate and improving the model nonlinear representation accuracy and its generalization ability. In the molecular encoding part, the addition of the residual network prevents the gradient from disappearing or exploding and ensures that the model can converge. In the extraction of molecular features, the involvement of the Transformer based on Double-head attention can focus on the features of the region of interest for the prediction results and assign the weights reasonably. Running our model on six datasets, our method outperformed current state-of-the-art methods in all metrics. The experimental results demonstrate the effectiveness of our proposed algorithmic framework.

## Data Availability

The dataset used in the experiments is provided by MoleculeNet and ChEMBL at http://ww82.moleculenet.ai/ and http://www.bioinf.jku.at/research/lsc/index.html. The codes and models are available at https://github.com/songyuanbing6/dhtnn.
